# Overexpression of geranylgeranyl diphosphate synthase contributes to tumour metastasis and correlates with poor prognosis of lung adenocarcinoma

**DOI:** 10.1111/jcmm.13493

**Published:** 2018-01-29

**Authors:** Xiaoxia Wang, Wujian Xu, Ping Zhan, Tianxiang Xu, Jiajia Jin, Yingying Miu, Zejun Zhou, Qingqing Zhu, Bing Wan, Guangmin Xi, Liang Ye, Yafang Liu, Jianwei Gao, Huijuan Li, Tangfeng Lv, Yong Song

**Affiliations:** ^1^ Department of Respiratory Medicine Jinling Hospital Southern Medical University Nanjing China; ^2^ Intensive Care Unit Inner Mongolia People's Hospital Hohhot China; ^3^ Center of Tumor Inner Mongolia People's Hospital Hohhot China

**Keywords:** geranylgeranyl diphosphate synthase, lung adenocarcinoma, prognosis, epithelial‐mesenchymal transition, metastasis, protein prenylation

## Abstract

This study aimed to evaluate the biological role of geranylgeranyl diphosphate synthase (GGPPS) in the progression of lung adenocarcinoma. GGPPS expression was detected in lung adenocarcinoma tissues by qRT‐PCR, tissue microarray (TMA) and western blotting. The relationships between GGPPS expression and the clinicopathological characteristics and prognosis of lung adenocarcinoma patients were assessed. GGPPS was down‐regulated in SPCA‐1, PC9 and A549 cells using siRNA and up‐regulated in A549 cells using an adenoviral vector. The biological roles of GGPPS in cell proliferation, apoptosis, migration and invasion were determined by MTT and colony formation assays, flow cytometry, and transwell and wound‐healing assays, respectively. In addition, the regulatory roles of GGPPS on the expression of several epithelial‐mesenchymal transition (EMT) markers were determined. Furthermore, the Rac1/Cdc42 prenylation was detected after knockdown of GGPPS in SPCA‐1 and PC9 cells. GGPPS expression was significantly increased in lung adenocarcinoma tissues compared to that in adjacent normal tissues. Overexpression of GGPPS was correlated with large tumours, high TNM stage, lymph node metastasis and poor prognosis in patients. Knockdown of GGPPS inhibited the migration and invasion of lung adenocarcinoma cells, but did not affect cell proliferation and apoptosis. Meanwhile, GGPPS inhibition significantly increased the expression of E‐cadherin and reduced the expression of N‐cadherin and vimentin in lung adenocarcinoma cells. In addition, the Rac1/Cdc42 geranylgeranylation was reduced by GGPPS knockdown. Overexpression of GGPPS correlates with poor prognosis of lung adenocarcinoma and contributes to metastasis through regulating EMT.

**Highlights**

1. GGPPS is overexpressed in lung adenocarcinoma.
2. Overexpression of GGPPS contributes to poor prognosis of lung adenocarcinoma.
3. Knockdown of GGPPS inhibits migration and invasion of lung adenocarcinoma cells.
4. GGPPS may promote metastasis of lung adenocarcinoma through regulating EMT.
5. Down‐regulation of GGPPS reduces the geranylgeranylation of Rac1/Cdc42.

## Introduction

Lung adenocarcinoma is a common and severe tumour that belongs to non‐small cell lung cancers (NSCLC). Although great advances have been made in the diagnosis and treatment of NSCLC, the worldwide mortality rate remains high 1. Patients with NSCLC often relapse and develop metastases after surgery, chemotherapy and/or radiation therapy, resulting in an overall five‐year survival rate of less than 18% 2. Researches into the molecular mechanisms underlying NSCLC progression and discovery of novel biomarkers and therapeutic targets have become hot topics in the clinic.

Geranylgeranyl diphosphate (GGPP) is an isoprenoid synthesized through the cellular mevalonate pathway 3. Defects in isoprenoid biosynthesis can be accompanied by various disease, including cancers, and metabolic and cardiovascular diseases 4. Geranylgeranyl diphosphate synthase (GGPPS) is an important branch‐point enzyme in the mevalonate metabolic pathway, which can synthesize GGPP from farnesyl diphosphate (FPP) 5, 6. Abnormal GGPPS expression can affect the relative levels of FPP and GGPP, participating in the regulation of many diseases such as cigarette smoke‐induced pulmonary disease 7, insulin resistance 8, 9, male infertility and cardiac hypertrophy 10, 11. Furthermore, digeranyl bisphosphonate inhibits GGPPS, resulting in the inhibition of protein geranylgeranylation in MDA‐MB‐231 cells, and contributing to the suppression of breast cancer cell migration 12. GGPPS is also important in the occurrence and progression of cirrhosis‐induced hepatocellular carcinoma (HCC) and can be used to predict the biological characteristics of HCC 13. However, related researches on the specific biological roles of GGPPS in lung adenocarcinoma are still limited.

In this study, GGPPS expression was detected in lung adenocarcinoma, and its relationships with clinicopathological characteristics and prognosis of patients were assessed. The regulatory roles of GGPPS on cell proliferation, apoptosis, migration and invasion of lung adenocarcinoma cells were analysed, and the regulatory mechanisms of GGPPS in EMT were further evaluated. Our findings may reveal the biological roles of GGPPS in the occurrence and progression of lung adenocarcinoma. GGPPS may thus be considered an independent biomarker for the prognosis of lung adenocarcinoma and a potential therapeutic target.

## Materials and methods

### Patients and tissue samples

A total of 32 adenocarcinoma tissues and adjacent normal tissues were collected from patients with lung adenocarcinoma. These patients had undergone surgical resection at the Department of Thoracic Surgery, Jinling Hospital, Nanjing, China, between July 2015 and February 2016. Patients who had received preoperative radiotherapy or chemotherapy were excluded from this study. All tissues were confirmed histopathologically as lung adenocarcinoma or adjacent normal lung tissue and stored in liquid nitrogen. This study was approved by the Institutional Review Board of Southern Medical University. Informed consents were obtained from all enrolled patients.

### Tissue microarray (TMA)

TMA (HLug‐Ade180Sur‐01, Shanghai Outdo Biotech, Shanghai, China) was used to evaluate GGPPS expression in 90 patients with lung adenocarcinoma who had undergone surgical resection between July 2004 and June 2009. These patients were followed up until August 2014. Among them, 5 patients were lost to follow up because of slide dropping. Antigen retrieval was performed by microwaving in 10 mmol/l sodium citrate buffer (pH 6) for 5 min., and tissues were incubated overnight with primary antibodies at 4°C (GGPS1 rabbit polyclonal antibody, 14944‐1‐AP; Proteintech, Chicago, USA). After incubation with a secondary antibody, images were collected and evaluated blindly by two independent pathologists. Staining intensity was scored as 0 (negative), 1 (weak), 2 (medium) or 3 (strong), and percentage scores of positive cells were defined as 0 (0%), 1 (1–25%), 2 (26–50%), 3 (51–75%) or 4 (76–100%). A staining index was calculated for each sample by multiplying the intensity and positivity scores (0–12).

### Cell culture

Lung adenocarcinoma cell lines, A549, SPC‐A1, PC9, H1975 and H1299 and a normal human bronchial epithelial cell line (HBE) were purchased from the Institute of Biochemistry and Cell Biology of the Chinese Academy of Sciences (Shanghai, China). A549, SPC‐A1, H1975 and H1299 were maintained in Roswell Park Memorial Institute (RPMI) 1640 basic medium (GIBCO‐BRL, Invitrogen, Carlsbad, CA) containing 10% foetal bovine serum (FBS), 100 U/l penicillin and 0.1 mg/ml streptomycin. PC9 and HBE cells were maintained in Dulbecco's modified Eagle's medium (DMEM) (GIBCO‐BRL, Invitrogen, Carlsbad, CA, USA) containing 10% foetal bovine serum (FBS), 100 U/l penicillin and 0.1 mg/ml streptomycin. All cells were cultured in a humidified incubator at 37°C with 5% CO_2_.

### qRT‐PCR

Total RNA was extracted from isolated tissues and cultured cells, using TRIzol reagent (Invitrogen). cDNA was reverse transcribed using a reverse transcription kit (Takara, Dalian, China). qRT‐PCR was carried out using SYBR Premix Ex Taq II (Perfect Real Time, TaKaRa) on an ABI 7500 real‐time PCR system (Applied Biosystems, Foster City, CA, USA). Specific primers were used as follows: GAPDH forward 5′GCAAATTCCATGGCACCGT3′, GAPDH reverse 5′GCCCCACTTGATTTTGGAGG3′; GGPPS forward 5′TGGAGAAGACTCAAGAAACAG′, GGPPS reverse 5′TCAGCCAATGATTAAATGCC′. GAPDH was used for normalization. PCR conditions were 94°C for 30 sec., followed by 40 cycles at 94°C for 5 sec. and 60°C for 34 sec. The relative expression level of GGPPS was calculated using the 2^−▵▵CT^ method.

### RNA interference by siRNA

The GGPPS siRNA sequence was amplified by PCR, using specific primers (GGPPS siRNA forward 5′GUCCCACUGAAGAAGAAUATT3′, GGPPS siRNA reverse 5′UAUUCUUCUUCAGUGGGACTT3′; Control siRNA forward 5′UUCUCCGAACGUGUCACGUTT3′, Control siRNA reverse 5′ACGUGACACGUUCGGAGAATT3′). When cells (SPC‐A1, PC9 and A549) reached 60–70% confluence, siRNA transfection was performed using Lipofectamine 2000 (Invitrogen, Shanghai, China). Forty‐eight hours after transfection, cells were used for qRT‐PCR and western blot analysis. To evaluate the recovery effects of exogenous GGPP, SPCA‐1 and PC9 cells transfected with siRNA‐GGPPS were treated with 20 μmol/l GGPP (Sigma‐Aldrich, diluted in methanol) for 24 hr 10, 11.

### Cell transfection with GGPPS adenovirus vector

GGPPS (Ad‐GGPPS) and control (Ad‐GFP) adenovirus vectors were kind gifts from Professor Chao‐jun Li 7. A549 cells were transfected when the cell density reached 50%. multiplicity of infection was 20. Green fluorescence was found in cells at 60 hr post‐transfection, and cells were harvested for qRT‐PCR and western blot analysis at 72 hr post‐transfection.

### MTT and colony formation assay

Cell viability of transfected cells was assessed every 24 hr by MTT, using a Cell Proliferation Reagent Kit I (Roche Applied Science Indianapolis, USA). A total of 750 transfected cells were plated onto six‐well plates with fresh medium containing 10% FBS. Medium was refreshed every 4 days. After 10–14 days of culturing, colonies were fixed with methanol, stained with 0.1% crystal violet (Sigma‐Aldrich, St. Louis, MO, USA) and manually counted.

### Flow cytometry

Apoptosis in transfected cells was analysed by flow cytometry (FACScan, BD Biosciences, San Jose, CA, USA) using CellQuest software (BD Biosciences). By staining with FITC‐annexin V and propidium iodide (PI), late apoptotic cells, early apoptotic cells, viable cells and dead cells were classified, and the ratio of apoptotic cells was calculated.

### Transwell migration and invasion assay

A total of 5 × 10^4^ cells and 1 × 10^5^ cells, grown in medium containing 1% FBS, were isolated for migration and invasion assays, respectively. These cells were seeded into the upper chamber of an insert (8‐μm pore size, Millipore). For invasion assays, the upper chamber of insert was pre‐coated with Matrigel (Corning, 356234). Medium containing 10% FBS was added to the lower chamber. After incubation at 37°C with 5% CO_2_ for 24 hr, cells in the upper layer were removed with cotton wool, and cells in lower chamber were fixed with methanol and stained with 0.1% crystal violet (Sigma‐Aldrich). Stained cells were photographed and counted in five random fields.

### Wound‐healing assay

A total of 5 × 10^4^ transfected cells were isolated for wound‐healing assay. After seeded onto dishes, a wound track was scored into each dish with a plastic scraper. Debris was removed by washing with PBS. After 0, 24 and 48 hr of culturing, photographs were captured using a microscope and the extent of wound healing was measured.

### HPLC‐MS/MS measurement of GGPP

A total of 5 × 10^6^ cells were harvested for the measurement of GGPP using HPLC‐MS/MS system as previously described 10. Simply, cell lysates were dried with nitrogen and resuspended in 100 μl Methanol:H_2_O (1:1, v:v). After 1 min. of centrifugation at 12,000 × *g*, the supernatants were transferred into clean tubes for HPLC‐MS analysis. The HPLC system consisted of two LC‐30A ultra‐high pressure gradient pumps, a vacuum degasser (DGU‐20A5) and an autosampler (SIL‐30AC). The samples were injected onto Inert Sustain C18 column (3 × 100 mm, 3 μm diameter, 20°C) and separated by a linear gradient between 0.1% NH_3_–H_2_O (A) and 100% acetonitrile (B) at a flow rate of 0.4 ml/min. The gradient was set as follows: 0–1 min.: 5% B to 5% B; 1–3 min.: 5% B to 80% B; 3–5 min.: 80% B to 80% B; 5–5.1 min.: 80% B to 5% B; 5.1–6.5 min.: equilibration with 5% B (total time 6.5 min.). MS (LCMS‐8050, Shimadzu, Japan) was performed in negative electrospray ionization mode using nitrogen as the nebulizing gas and argon as the collision gas. Finally, GGPP was detected with the mass spectrometer (MS) in Multiple Reaction Monitoring (MRM) mode (parent ion *m*/*z*: 449.15 to product ion *m*/*z*: 79.20). Total GGPP level (ng/mg protein) was normalized to total protein contents, as measured by BCA assay.

### Prenylation and membrane association measurements

Protein prenylation was measured as described previously 8. In brief, SPCA‐1 and PC9 cells with the indicated treatment lysed in 500 μl lysis buffer. The total protein concentration was diluted to 1 mg/ml and partitioned with same volume of 4% Triton X‐114 for 5 min. at 37°C to solubilize and fractionate the lipid‐rich cell membrane. The aqueous upper phase contains the water‐soluble small GTPases, and the organic lower phase contains the lipid‐soluble small GTPases. The membrane proteins of transfected cells were extracted using Mem‐PER Plus Membrane Protein Extraction Kit (Thermo Scientific, 89842). Simply, 5 × 10^6^ cells were harvested by 5 min. of centrifugation at 300 × *g* and washed with 3 ml cell wash solution. About 0.75 ml permeabilization buffer was added into cell depositions and incubated for 10 min. at 4°C with constant mixing. After 15 min. of centrifugation at 16,000 × *g*, the supernatant containing cytosolic proteins was collected. Then, 0.5 ml solubilization buffer was added into cell depositions and incubated for 30 min. at 4°C with constant mixing. After 15 min. of centrifugation at 16,000 × *g*, supernatant containing solubilized membrane and membrane‐associated proteins was collected. The subcellular fractions were immunoprecipitated with Rac1/Cdc42 antibody (Cell Signaling Technology, Danvers, MA, USA), and subsequently subjected to western blot analysis.

### Immunoprecipitation

Immunoprecipitation was performed according to a standard protocol 14. Rac1/Cdc42 antibody was used to form immune complex with Rac1/Cdc42 proteins in lysates and was immunoprecipitated down with protein A/G plus agarose. Finally, the equivalent protein samples were subjected to Western blot analysis against Rac1/Cdc42.

### Western blot assay

Total proteins were isolated from tissues and cells, using lysis buffer containing mammalian protein extraction reagent RIPA (Beyotime, China), PMSF (Roche) and protease inhibitor cocktail (Roche, Basel, Switzerland). Protein concentration was determined using a Bio‐Rad Protein Assay kit (KeyGEN Biotech, Jiangsu, China). Protein samples (30 μg) were electrophoresed by 12% or 10% SDS‐PAGE, transferred to 0.22 μm polyvinylidene fluoride (PVDF) membranes (Millipore, USA) and incubated with specific antibodies (GGPPS, 1:500, sc‐271680, Santa Cruz Biotechnology, TX, USA; GAPDH, 1:10,000, ab181602, Abcam, Cambridge, UK; E‐Cadherin, 1:1000, 3195, Cell Signaling Technology; N‐Cadherin, 1:1000, 13116, Cell Signaling Technology; Vimentin, 1:1000, 5741, Cell Signaling Technology; Rac1/Cdc42,1:1000,4651, Cell Signaling Technology). After incubation with secondary antibody, protein bands were detected using an ECL detection system (Tanon, China).

### Statistical analysis

All experiments were performed in triplicate, and data were expressed as mean ± standard deviation (S.D.). All statistical analyses were performed using SPSS 20.0 software (IBM, Chicago, IL, USA). An independent sample *t*‐test was performed to compare the mean values of different groups. A chi‐square test was performed to evaluate the correlation between GGPPS and the clinical characteristics of patients. Kaplan–Meier survival analysis was performed to assess the correlation between GGPPS and overall survival of patients. A Cox regression model was used to analyse independent prognostic risk factors. A *P*‐value less than 0.05 was considered significantly different.

## Results

### GGPPS is up‐regulated in lung adenocarcinoma

**Figure 1:**
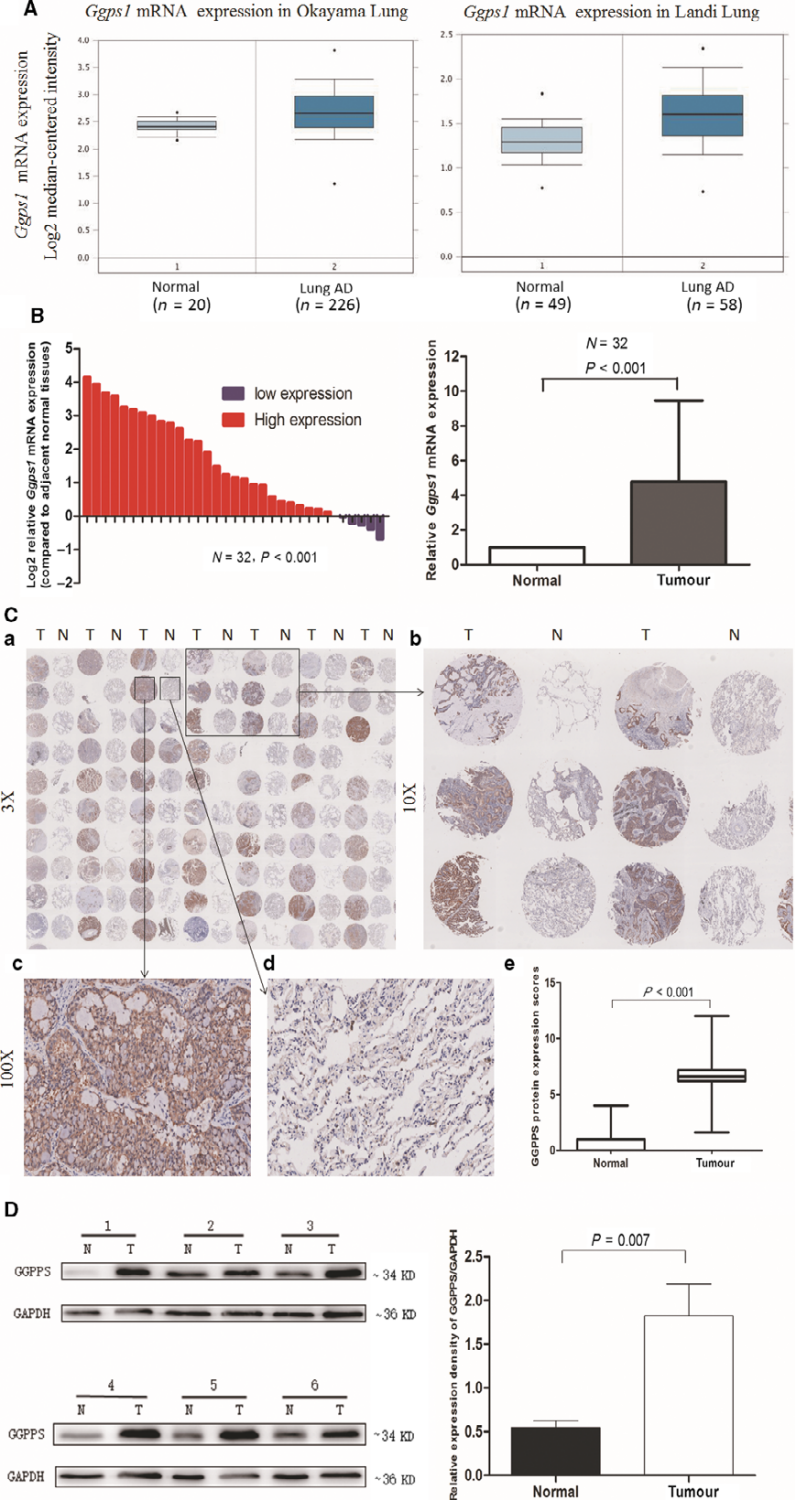
GGPPS expression was increased in lung adenocarcinoma tissues at both the mRNA and protein level. (**A**) Increased expression of *Ggps1* mRNA from two studies from the Oncomine database. (**B**) qRT‐PCR analysis of relative *Ggps1* mRNA expression in lung adenocarcinoma and adjacent normal tissues (*n* = 32). (**C**) a. Tissue microarray (TMA) of GGPPS protein in 90 lung adenocarcinoma tissues and adjacent normal tissues. b. Tissue microarray (TMA) of GGPPS protein in six pairs of tissues. c. High GGPPS expression in lung adenocarcinoma tissue. d. Low GGPPS expression in lung adenocarcinoma tissues. e. Expression scores of GGPPS protein in lung adenocarcinoma and adjacent normal tissues. (**D**) Western blot analysis of GGPPS expression in lung adenocarcinoma (*n* = 6) and adjacent normal tissues (*n* = 6). Quantitative analysis of relative expression density is shown in the right panel (T: tumour; N: non‐tumour).

The expression of GGPPS has been reported to be significantly higher in lung adenocarcinoma tissues than in normal lung tissues 15, 16, 17, 18, 19 (Figs 1A and S1). In consistent with these findings, we found that *Ggps1* mRNA expression was significantly higher in lung adenocarcinoma tissues than in adjacent normal tissues (*P* < 0.001, Fig. 1B). At the protein level, TMA showed a significantly higher GGPPS content in lung adenocarcinoma tissues than in adjacent normal tissues (*P* < 0.001, Fig. 1C). Furthermore, the increased GGPPS protein expression in lung adenocarcinoma tissues was also confirmed by western blotting (*P* < 0.01, Fig. 1D).

### Relationship between GGPPS expression and the clinicopathological characteristics and prognosis of patients with lung adenocarcinoma

**Figure 2:**
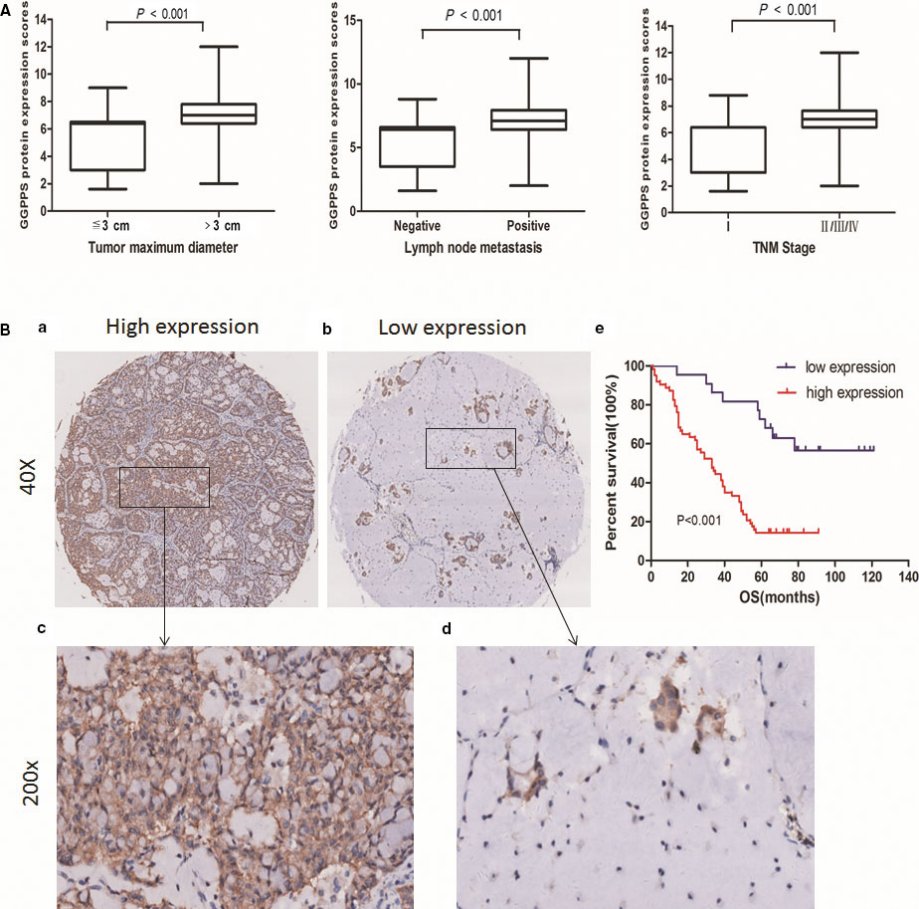
Associations between GGPPS expression and clinicopathological characteristics and prognosis of patients with lung adenocarcinoma. (**A**) High GGPPS expression in patients with large tumours, lymph node metastasis and advanced TNM stages. (**B**) GGPPS overexpression was accompanied with poor overall survival in patients with lung adenocarcinomas. (a, c) High GGPPS expression in lung adenocarcinomas (≥6.4). (b, d) Low GGPPS expression in lung adenocarcinomas (<6.4). (e) Kaplan–Meier survival analysis showed that high GGPPS expression correlates with decreased overall survival (log‐rank test, *P *<* *0.001).

The relationship between GGPPS expression (staining score) and the clinicopathological parameters of patients was assessed. The results showed that the increased GGPPS expression in lung adenocarcinoma was significantly associated with large tumour size (>3 cm, *P* < 0.05), lymph node metastasis (*P *< 0.001) and high TNM stage (*P *< 0.001, Fig. 2A) (Table 1). The 85 patients could be divided into high‐expression and low‐expression groups, according to GGPPS expression levels, using 6.4 as a cut‐off value (<6.4) (Fig. 2B).

**Table 1:** Correlation between GGPPS protein expression and clinicopathological parameters of lung adenocarcinoma patients

Characteristics	Number of patients	GGPPS protein expression		
		Low <6.4	High ≧6.4	*P*‐value†
All patients	85	22	63	
Gender				
Male	45	9	36	0.189
Female	40	13	27	
Age				
<65 years	47	13	34	0.677
≧65 years	38	9	29	
Size of tumour				
≦3 cm	33	13	20	0.023*
>3 cm	52	9	43	
Lymph node metastasis (pN)				
N0	37	16	21	0.001*
N1–3	48	6	42	
p‐TNM stages				
I (Ia, Ib)	27	13	14	0.001*
II–IV	58	9	49	

*P < 0.05. ^†^Chi‐square test.

The relationship between GGPPS expression and patient prognosis was further analysed by Kaplan–Meier survival analysis. As shown in Fig. 2Be, patients with high expression levels of GGPPS exhibited a significantly shorter OS than those with low expression levels (*P* < 0.001, Fig. 2Be). Furthermore, Cox regression analysis confirmed that high GGPPS expression contributed to the poor prognosis of lung adenocarcinoma (HR = 3.539, 95% CI: 1.652–7.581) (Table 2).

**Table 2:** Cox regression analysis of GGPPS protein expression and other clinical prognostic factors for overall survival in patients with lung adenocarcinoma (*n* = 85)

Factors	HR	Univariate analysis 95%CI	*P*	HR	Multivariate analysis 95%CI	*P*
Gender (Female/Male)	0.786	0.478–1.294	0.344	0.891	0.533–1.490	0.661
Age (≥65/<65 years)	1.080	0.658–1.774	0.761	1.060	0.642–1.751	0.820
Size of tumour (>3 cm/≤3 cm)	1.592	0.938–2.702	0.085	1.146	0.662–1.984	0.627
Lymph node metastasis (N1–3/N0)	2.654	1.556–4.525	0.000*	1.796	1.029–3.135	0.039*
p‐TNM stages (II + III + IV/I)	2.519	1.398–4.538	0.002*	1.163	0.437–3.096	0.762
GGPPS expression (high/low)	4.510	2.183–9.316	0.000*	3.539	1.652–7.581	0.001*

HR: hazard ration; 95% CI: 95% confidence interval. *P < 0.05.

### Biological function of GGPPS in lung adenocarcinoma cells

**Figure 3:**
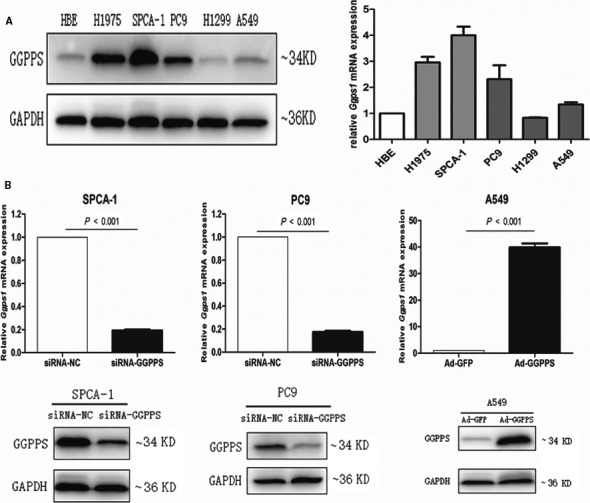
GGPPS expression in cells transfected with siRNA‐GGPPS and adenoviral vector Ad‐GGPPS. (**A**) Western blot and qRT‐PCR analysis of GGPPS expression in five lung adenocarcinoma cell lines (H1975, SPCA‐1, PC9, H1299 and A549) and one human bronchial epithelial cell (HBE). (**B**) qRT‐PCR and western blot analysis of GGPPS expression in SPCA‐1 and PC9 cells transfected with siRNA‐GGPPS and A549 cells transfected with adenoviral vector Ad‐GGPPS.

To evaluate the biological function of GGPPS in lung adenocarcinoma cells, GGPPS expression levels were measured in five NSCLC cell lines and HBE cells. Results showed that the expression of GGPPS was relatively higher in SPCA‐1, H1975 and PC9 cells, slightly higher in A549, but relatively lower in H1299 than in HBE cells (Fig. 3A).

**Figure 4:**
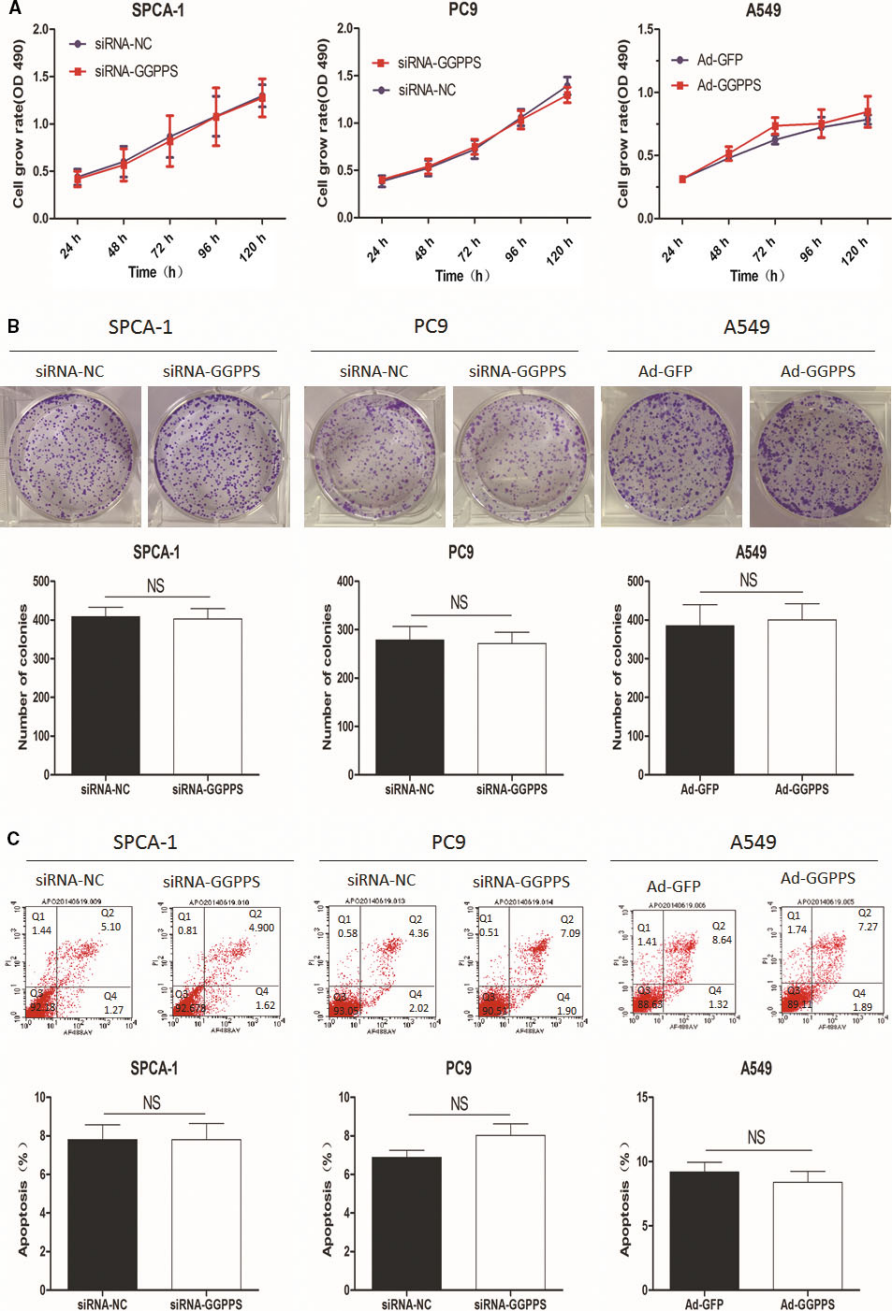
Regulatory roles of GGPPS in the proliferation and apoptosis of SPCA‐1 and PC9 cells transfected with siRNA‐GGPPS, and A549 cells transfected with adenoviral vector Ad‐GGPPS. (**A**) Cell growth rates. (**B**) Number of colonies. (**C**) Apoptotic rates.

**Figure 5:**
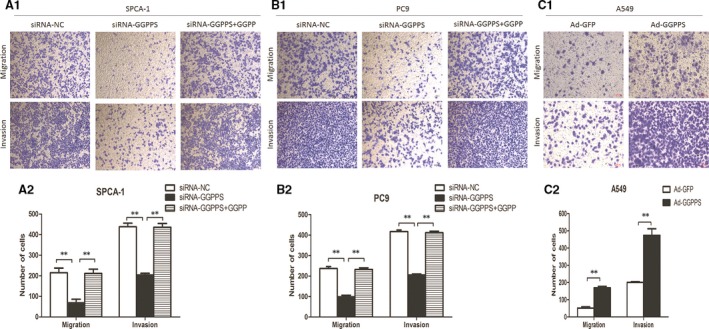
Regulatory roles of GGPPS in the migration and invasion of SPCA‐1 and PC9 cells transfected with siRNA‐GGPPS, and A549 cells transfected with adenoviral vector Ad‐GGPPS. (**A1, B1**) Transwell migration and invasion assays showed that the migration and invasion of lung adenocarcinoma cells were inhibited by reduced GGPPS expression (SPCA‐1 and PC9 cells), and the changes were recovered by the administration of 20 μmol/l GGPP for 24 hr. (**C1**) Transwell assays showed that up‐regulation of GGPPS promoted the migration and invasion of A549 cells. (**A2, B2, C2**). The statistical analysis graphs of A1,B1,C1. **P < 0.01.

GGPPS was down‐regulated in SPCA‐1, PC9 and A549 cells using siRNA and up‐regulated in A549 cells using adenoviral vector Ad‐GGPPS (*P* < 0.001, Figs 3B and S2A). The effects of GGPPS expression on cell proliferation were determined by MTT and colony formation assays. However, neither down‐regulation nor up‐regulation of GGPPS influenced the growth of SPCA1, PC9 and A549 cells (Figs 4A and B; Fig. S2B,C). In addition, flow cytometry showed that the changed expression of GGPPS also did not affect the apoptosis of those cells (Figs 4C and S2D). However, transwell assay revealed that the down‐regulation of GGPPS significantly inhibited the migration and invasion of lung adenocarcinoma cells (SPCA‐1, PC9 and A549 cells), and up‐regulation of GGPPS significantly increased the migration and invasion of A549 cells (*P* < 0.01, Figs 5 and S3D). Importantly, GGPP administration recovered the inhibited migration and invasion of SPCA‐1 and PC9 cells transfected with siRNA‐GGPPS (*P* < 0.01, Fig. 5A1, B1, A2, B2). Moreover, the inhibited migration induced by GGPPS down‐regulation was also identified in SPCA‐1, PC9 and A549 cells by wound‐healing assay (*P* < 0.01, Fig. S3A, B, C).

### Mechanism underlying the regulatory role of GGPPS in the migration and invasion of lung adenocarcinoma cells

**Figure 6:**
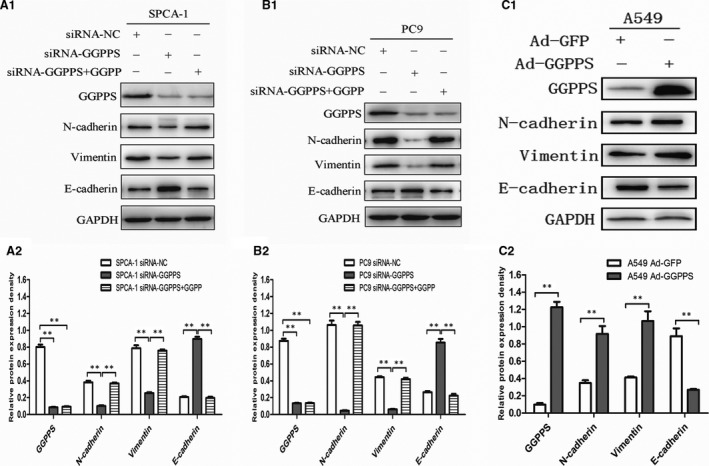
Regulatory role of GGPPS in metastasis of lung adenocarcinoma cells was associated with EMT. (**A1, B1**) Effects of down‐regulated GGPPS on the expression of several key EMT markers in SPCA‐1, PC9 cells, and the effects were recovered by the administration of 20 μmol/l GGPP for 24 hr. (**C1**) Effects of up‐regulated GGPPS on the expression of several key EMT markers in A549 cells. (**A2, B2, C2**) Quantitative analysis of relative expression levels. **P < 0.01.

EMT is a key mechanism underlying the migration and invasion of tumour cells 20, 21. Thus, we assayed the expression of several important EMT markers in GGPPS downexpressed SPCA‐1, PC9, A549 cells and in GGPPS overexpressed A549 cells. As shown in Figs 6 and S4, the expression of E‐cadherin was significantly increased in GGPPS downexpressed SPCA‐1, PC9, A549 cells, while the expression of N‐cadherin and vimentin was reduced (*P* < 0.01, Figs 6A1, B1, A2, B2 and S4). On the contrary, the expression of E‐cadherin in GGPPS overexpressed A549 cells was significantly decreased, and the expression of N‐cadherin and vimentin was increased (*P* < 0.01, Fig. 6C1, C2). Besides, GGPP administration recovered the expression of E‐cadherin, N‐cadherin and vimentin in SPCA‐1 and PC9 cells transfected with siRNA‐GGPPS (*P* < 0.01, Fig. 6A1, B1, A2, B2).

### Down‐regulation of GGPPS in SPCA‐1 and PC9 cells reduced Rac1/Cdc42 geranylgeranylation

**Figure 7:**
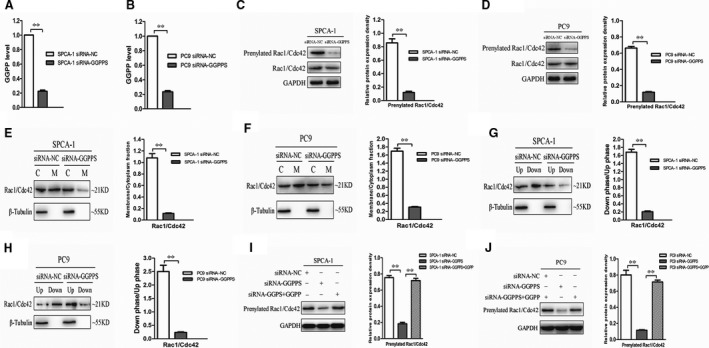
Protein prenylation was altered by down‐regulated GGPPS in SPCA‐1 and PC9 cells. (**A, B**) HPLC‐MS/MS detection showed that the level of GGPP(ng/mg protein) significantly decreased after knockdown of GGPPS in SPCA‐1 and PC9 cells. **P < 0.01. (**C, D**) The Rac1/Cdc42 prenylation was decreased after knockdown of GGPPS in SPCA‐1 and PC9 cells. **P < 0.01. (**E, F**) The membrane association of Rac1/Cdc42 was decreased after GGPPS knockdown in SPCA‐1 and PC9 cells. **P < 0.01. (**G, H**) The lower/upper phase of Rac1/Cdc42 was decreased in SPCA‐1 and PC9 cells transfected with siRNA‐GGPPS. **P < 0.01. (**I, J**) The decreased Rac1/Cdc42 prenylation in SPCA‐1 and PC9 cells transfected with siRNA‐GGPPS was recovered by the administration of 20 μmol/l GGPP for 24 hr. **P < 0.01.

Previous studies have indicated that GGPPS deletion can reduce the content of GGPP and increase the content of FPP 10, 11. To determine whether protein prenylation was disrupted by downexpressed GGPPS in lung adenocarcinoma cells, the level of GGPP was detected. The HPLC‐MS/MS showed that the GGPP level was significantly decreased in SPCA‐1 and PC9 cells transfected with siRNA‐GGPPS (*P* < 0.01, Fig. 7A and B). The Rho GTPases Rac1 and Cdc42, representative geranylgeranylated proteins, have been reported to be able to regulate cell migration 22, 23, 24. We examined the hydrophobicity and membrane association of Rac1/Cdc42 in SPCA‐1 and PC9 cells transfected with siRNA‐GGPPS. The results showed the Rac1/Cdc42 geranylgeranylation was significantly reduced in GGPPS downexpressed SPCA‐1 and PC9 cells (*P* < 0.01, Fig. 7C and D). The Rac1/Cdc42 membrane association and its expression in organic phase were significantly decreased (*P* < 0.01, Fig. 7E–H). Further analysis revealed that the decreased Rac1/Cdc42 prenylation following knockdown of GGPPS in SPCA‐1 and PC9 cells was recovered with GGPP administration (*P* < 0.01, Fig. 7I and J).

## Discussion

Protein prenylation is an important post‐translational protein modification that is critical for the membrane association of a number of signalling proteins involved in cell differentiation, proliferation and migration 25. A series of signalling pathways will be altered once this modification is disrupted, which accounts for the pathological progression of many diseases including neurodegenerative disorders and cancers 4. The mevalonate pathway is considered as a target in the treatment of cancers 26, 27, 28. It has been reported that geranylgeranyl transferase inhibitors (GGTIs) can act as anti‐cancer agents by inhibiting the proliferation and invasion of cancer cells 29, 30, 31. Statins (HMG‐CoA reductase inhibitor) and bisphosphonates (farnesyl diphosphate synthase inhibitor) have been shown to promote apoptosis and inhibit cancer cell migration 32, 33, while the anti‐cancer effects of statins and bisphosphonates are inhibited by GGPP 34, 35, 36. Geranylgeranyl diphosphate synthase (GGPPS) is an important branch‐point enzyme in the mevalonate metabolic pathway and participates in regulating the protein prenylation. Therefore, it is worthy to study the biological role of GGPPS in cancer, and whether GGPPS can serve as a target in the treatment of cancer.

In the present study, significantly higher GGPPS expression was found in lung adenocarcinoma tissues than in adjacent normal tissues. This result is consistent with that of previous research 13, and further identifies an important role for GGPPS in lung adenocarcinoma. GGPPS up‐regulation was associated with increased tumour size, lymph node metastasis, advanced TNM stage and poor prognosis of lung adenocarcinoma. These results indicate that GGPPS is closely related to tumour development and may thus be used as a biomarker and therapeutic target in lung adenocarcinoma.

GGPPS inhibition can reduce RhoA geranylgeranylation and membrane localization through GGPP depletion, and thus inhibit breast cancer cell migration 12. Previous studies have indicated that GGPPS knockout altered protein prenylation in Sertoli cells and Neonatal rat ventricular myocytes (NRVMs) 10, 11. In the present study, we found that the Rac1/Cdc42 geranylgeranylation was decreased by knockdown of GGPPS, and the GGPP administration could recover the changes. Furthermore, GGPPS down‐regulation inhibited the migration and invasion of lung adenocarcinoma cells, and GGPPS up‐regulation significantly increased the migration and invasion of A549 cells. However, the proliferation and apoptosis of lung adenocarcinoma cells were not affected. These results may suggest that GGPPS down‐regulation reduces prenylation and membrane association of small GTPase protein which regulates cell migration, thereby inhibiting the migration and invasion of lung adenocarcinoma cells.

The molecular mechanisms of GGPPS in the migration and invasion of lung adenocarcinoma cells were further evaluated in the present study. E‐cadherin expression was significantly increased following knockdown of GGPPS, while the expression of N‐cadherin and vimentin was reduced. In addition, the expression of E‐cadherin in GGPPS overexpressed A549 cells was significantly decreased, while the expression of N‐cadherin and vimentin was increased. E‐cadherin, N‐cadherin and vimentin are well‐known markers of EMT 37, 38, 39, which is a vital process in the invasion and metastasis of tumour cells 40, 41. Our study demonstrated for the first time that GGPPS contributes to the migration and invasion of lung adenocarcinoma cells, at least partially through the regulation of EMT. Additionally, the Rho GTPases, Rac1 and Cdc42 are known to regulate cell migration, which are related to lung cancer metastasis 22, 23, 24, 42. These Rho GTPases are preferentially geranylgeranylated by GGPP through GGTase I 43. We found that Rac1/Cdc42 geranylgeranylation was reduced by GGPPS knockdown. This results suggest that GGPPS down‐regulation may reduce Rho GTPase geranylgeranylation and inhibit downstream signalling pathways related to EMT 44, thereby inhibiting tumour cell metastasis. In addition, the recovery effects of exogenous GGPP indicate that GGPPS knockdown is contribute to enzyme deactivation in GGPP production.

## Conclusion

In conclusion, GGPPS is overexpressed in lung adenocarcinoma, and thus may function as a prognostic factor. Knockdown of GGPPS may inhibit the migration and invasion of lung adenocarcinoma through EMT regulation. However, the precise signalling pathways responsible for the biological functions of GGPPS in EMT still need to be studied. Further researches on the action mechanisms of GGPPS in lung adenocarcinoma are needed.

## Conflict of interests

All authors declare that they have no conflict of interests to state.

## Supporting information


**Fig. S1** *Ggps1* mRNA expression was significantly increased in lung adenocarcinoma tissues compared to that in normal lung tissues in three studies. Data were obtained from the publicly available database Oncomine.

**Fig. S2** Regulatory roles of GGPPS in the proliferation and apoptosis of A549 cells. (A) qRT‐PCR and western blot analysis of GGPPS expression in A549 cells transfected with siRNA‐GGPPS. (B) Cell growth rates. (C) Number of colonies. (C) Apoptotic rates.

**Fig. S3** Down‐regulation of GGPPS inhibits migration of lung adenocarcinoma cells. (A,B,C) Wound‐healing assay showed that the migration rates of lung adenocarcinoma cells (SPCA‐1, PC9, A549) transfected with siRNA‐GGPPS were lower than those transfected with siRNA‐control. (D) Transwell assay showed that GGPPS knockdown reduced the migration and invasion of A549 cells.

**Fig. S4** Effects of GGPPS knockdown on the expression of several key EMT markers in A549 cells. (A) Western blotting showed that the down‐regulated GGPPS significantly reduced the expression of N‐cadherin and Vimentin, but increased the expression of E‐cadherin in A549 cells. (B) Quantitative analysis of relative proteins expression density. **P < 0.01.
